# Clozapine in pregnancy

**DOI:** 10.4103/0019-5545.31586

**Published:** 2006

**Authors:** Sujata Sethi

**Affiliations:** * Lecturer, Department of Psychiatry, PGIMS, Rohtak; *mailing address:* 122/8, Shivaji Colony, Rohtak 124001, Haryana; e-mail: reachsujata@rediffmail.com

**Keywords:** Clozapine, pregnancy

## Abstract

This report describes the case of a woman with treatment-resistant schizophrenia who became pregnant while being treated successfully with clozapine. Possible risks associated with continuation of clozapine during pregnancy are discussed.

## INTRODUCTION

Increasing use of clozapine in the treatment of psychotic illness has associated caveats. One of these being increased chances of pregnancy both desired and unwanted in woman taking clozapine. Sufficient data are not available to psychiatrists to guide the management of such cases. The following case adds to the existing data on the use of clozapine during pregnancy.

## THE CASE

Ms S, a 20-year-old unmarried woman from middle socio-economic status, presented with a history of 2 years' duration suggestive of schizophrenia according to the DSM-IV guidelines. She had been treated with various typical and atypical antipsychotics. She showed no improvement but experienced side-effects. So treatment with clozapine was considered. Under regular leucocyte monitoring, the dose of clozapine was gradually increased to a maximum of 250 mg/day. She showed remarkable improvement and got married in the next 6 months. Despite repeated advice to practice contraception, Ms S disclosed about being pregnant at the end of the first trimester. She and her husband were counselled on the possible harm to the foetus by clozapine, but they decided to continue with the pregnancy as well as with the treatment at the same dose fearing that she might relapse with dose reduction. Besides regular antenatal check-up, blood sugar and leucocyte counts were monitored regularly. She delivered a healthy female child without any complications. She was advised not to breastfeed. The child has been followed up for 2 years and has shown no developmental delays. The patient is also maintaining well.

## DISCUSSION

Data regarding the potential teratogenicity of clozapine are not unanimous. There are reports of no congenital abnormalities with clozapine in animals and human beings.^1^ In contrast, Dev and Krupp reviewed the literature and reported congenital malformations in 10 children and perinatal syndromes in 61 children exposed to clozapine during pregnancy.^2^ Another report described intrauterine foetal death in a woman taking clozapine but it is not clear whether it was clozapine only or a combination of other factors which contributed to this outcome.^3^ Increased glucose intolerance during pregnancy,^4^ precipitation of gestational diabetes with shoulder dystocia^5^,^6^ have been reported in patients taking clozapine.

However, in our patient clozapine was continued at the same dose throughout the gestational period with no hyperglycaelmia or any complication during labour and delivery. The child also showed no congenital anomaly. Breastfeeding was not advised considering the high concentration of clozapine in breast milk and exposing the child further to clozapine.^7^

Since the introduction of atypical antipsychotics particularly clozapine, increasing number of patients with treatment-resistant schizophrenia are benefiting in terms of remission and better social functioning. Unlike typical antipsychotics, clozapine does not cause hyperprolactinaemia, thus increasing the chances of a woman taking clozapine to conceive. Though the patient described here had an uneventful pregnancy while on clozapine, it needs to be emphasized that like other psychotropics clozapine should be used with caution during pregnancy. This is more so with clozapine as there is insufficient knowledge regarding clozapine-induced agranulo-cytosis in foetus/neonate.
